# Forgotten Actors: Glycoside Hydrolases During Elongation Growth of Maize Primary Root

**DOI:** 10.3389/fpls.2021.802424

**Published:** 2022-02-10

**Authors:** Alsu Nazipova, Oleg Gorshkov, Elena Eneyskaya, Natalia Petrova, Anna Kulminskaya, Tatyana Gorshkova, Liudmila Kozlova

**Affiliations:** ^1^Kazan Institute of Biochemistry and Biophysics, FRC Kazan Scientific Center of RAS, Kazan, Russia; ^2^Petersburg Nuclear Physics Institute Named by B.P. Konstantinov of National Research Center “Kurchatov Institute”, Gatchina, Russia; ^3^Kurchatov Genome Center - PNPI, Gatchina, Russia

**Keywords:** cell wall, elongation (growth), maize (*Zea mays* L.), root, glycoside hydrolase, RNA-seq

## Abstract

Plant cell enlargement is coupled to dynamic changes in cell wall composition and properties. Such rearrangements are provided, besides the differential synthesis of individual cell wall components, by enzymes that modify polysaccharides *in muro*. To reveal enzymes that may contribute to these modifications and relate them to stages of elongation growth in grasses, we carried out a transcriptomic study of five zones of the primary maize root. In the initiation of elongation, significant changes occur with xyloglucan: once synthesized in the meristem, it can be linked to other polysaccharides through the action of hetero-specific xyloglucan endotransglycosidases, whose expression boosts at this stage. Later, genes for xyloglucan hydrolases are upregulated. Two different sets of enzymes capable of modifying glucuronoarabinoxylans, mainly bifunctional α-arabinofuranosidases/β-xylosidases and β-xylanases, are expressed in the maize root to treat the xylans of primary and secondary cell walls, respectively. The first set is highly pronounced in the stage of active elongation, while the second is at elongation termination. Genes encoding several glycoside hydrolases that are able to degrade mixed-linkage glucan are downregulated specifically at the active elongation. It indicates the significance of mixed-linkage glucans for the cell elongation process. The possibility that many glycoside hydrolases act as transglycosylases *in muro* is discussed.

## Introduction

Elongation or expansion growth of plant cells generally occurs between their division and specialization. During this process, they irreversibly elongate or expand many times compared to the meristematic initial (Cosgrove, 2005). Only cells that are surrounded by thin primary cell walls are able to increase their size, so primary cell walls have to be extensible and strong enough to withstand turgor pressure. Primary cell walls are mainly composed of polysaccharides and are classified into two types in angiosperms (Carpita, 1996). The dicots and non-commelinid monocots possess type I cell walls in contrast to type II cell walls of commelinid monocots including *Poales*. Cellulose is common for both types; xyloglucans (XyGs) and pectins are the basic matrix polysaccharides for cell walls of type I, and glucuronoarabinoxylans (GAXs) and mixed-linkage glucans (MLGs) are the main hemicelluloses in cell walls of type II (Carpita, 1996).

**FIGURE 1:**
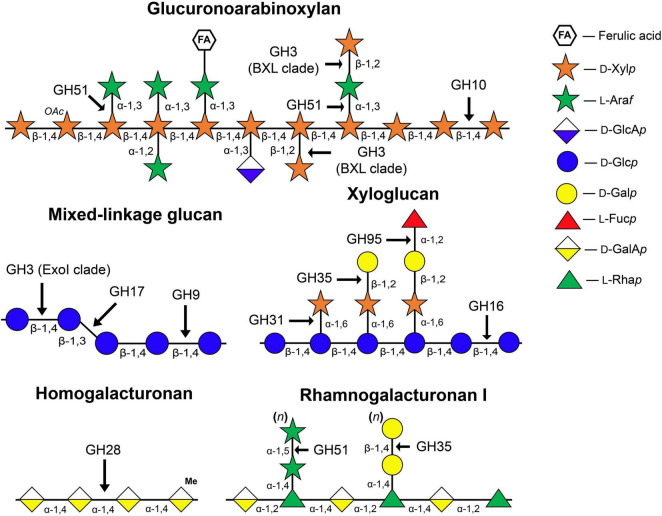
Structure of matrix polysaccharides typical for primary cell walls and enzyme families whose members catalyze the cleavage or modification of glycosidic linkages indicated by arrows. The symbolic representation of monosaccharides is according to GlycoPedia (Pérez, 2014) https://www.glycopedia.eu. GH, glycoside hydrolase.

One of the early hypotheses of the cell wall expansion mechanism was that glycoside hydrolases (GHs) act on so-called load-bearing linkages within the cellulose-hemicellulose network, so as to promote an increase in cell size. This point of view was supported by several observations: (i) Cell walls are capable of autolysis. During growth, the cell walls of both dicots and monocots lose some sugars, mainly glucose (Matchett and Nance, 1962; Wada et al., 1968); (ii) Extracts of growing tissues show enzymatic activities toward hemicelluloses (Davies and Maclachlan, 1968; Johnson et al., 1974; Huber and Nevins, 1980); (iii) The elongation growth of maize coleoptiles induced by auxin is inhibited by the infiltration of polyclonal antibodies specific to endo- and exo-β-D-glucanases (Hoson and Nevins, 1989; Inouhe and Nevins, 1991). The schematic structure of matrix polysaccharides of primary cell walls and GH families that can modify these polymers are represented in Figure 1.

However, after the discovery of expansins (McQueen-Mason et al., 1992), the significance of plant GHs in the elongation process was subjected to a question. At present, GHs are thought to participate in cell wall polysaccharide turnover; although neither cause nor effect of this intensive metabolism is understood yet (Barnes and Anderson, 2018). For instance, MLG turnover is coupled to the day and night cycle in maize leaves *via* the expression of an MLG-specific hydrolase (Kraemer et al., 2021). GHs take part in cellulose microfibril formation (Nicol et al., 1998; Lane et al., 2001; Szyjanowicz et al., 2004) and control the angle of cellulose microfibrils (Derba-Maceluch et al., 2015). The importance of GHs in different physiological processes such as root aerenchyma formation (Grandis et al., 2019), proper cell wall deposition (Moneo-Sánchez et al., 2019, 2020), and xylem differentiation (Yu et al., 2013; Hu et al., 2020; Tu et al., 2020) was shown. Despite a wide variety of GHs acting during different processes has been described in several omics studies (Francin-Allami et al., 2015; Calderan-Rodrigues et al., 2017; Grandis et al., 2019), in many cases, the physiological significance of these GHs in plants has remained elusive.

Here, we report a comprehensive characterization of cell wall-related GHs in the maize genome. All genes encoding the representatives of these protein families were identified and characterized. Transcriptomics analysis of their expression, together with enzymatic activity determination in the apical zones of growing maize root, permitted to advance the understanding of occurring cell wall rearrangements and their timing through the stages of root cell development.

## Materials and Methods

### Plant Material

**FIGURE 2:**
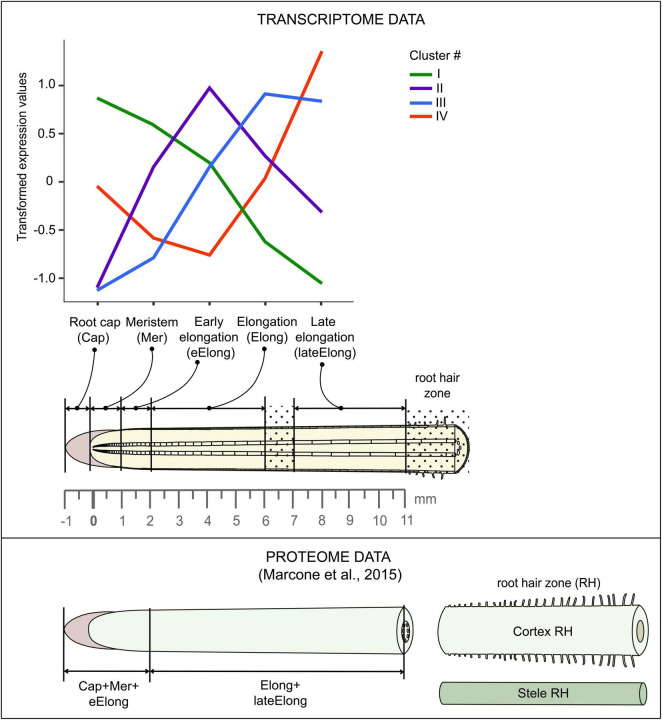
Scheme of maize root sampling for transcriptome and proteome (Marcon et al., 2015) analyses and four clusters of GH transcript abundance obtained by cluster analysis. The dot painting indicates maize root zones that were not used for analyses in this study.

Primary roots of 4-day old maize (*Zea mays* L., cv Mashuk) seedlings were used for this study. Maize grains were sterilized by 10-min incubation in 1% sodium hypochlorite solution and then washed three times in distilled water for 10 min. The seedlings were grown hydroponically in the dark at 27°C in distilled water. The primary root was subdivided into zones according to the following pattern: root cap, meristem (0-1 mm from the root cap junction), early elongation (1-2 mm), elongation (2-6 mm), and late elongation (7-11 mm), as described in Kozlova et al. (2020) and (Figure 2).

### Glycoside Hydrolase Activity and Protein Content Assay

Primary roots from 30 to 40 plants were cut into the studied zones (Figure 2), and samples were fixed in liquid nitrogen. The plant material was ground using mortar and pestle in a 50-mM NaOAc buffer (pH 5.6) with 0.05% (w/w) sodium azide and 3 mM dithiothreitol at 4°C. Homogenates were clarified by 10-min centrifugation at 5,000 *g* and used for enzyme activity and protein content (Bradford, 1976) assays. Enzyme activities were measured using the following substrates: *p*-nitrophenyl β-D-glucopyranoside, *p*-nitrophenyl β-D-galactopyranoside, *p*-nitrophenyl β-D-xylopyranoside, *p*-nitrophenyl α-D-mannopyranoside, *p*-nitrophenyl-α-L-fucopyranoside, and β-N-acetyl glucopyranoside at 10 mM concentration. Reaction mixtures containing 20-50 μl of clarified homogenates and 50 μl of a substrate solution were incubated at 37°C in a water bath for 10 min – 1 h depending on the reaction rate. The reaction was stopped by adding 900 μl of 10% (w/w) Na_2_CO_3_ solution. The optical density at 400 nm was determined on a spectrophotometer (V-576, Jasco, Tokyo, Japan). The amount of 4-nitrophenol liberated as a result of glycosidase catalysis was determined using a reference extinction coefficient (Dawson, 1986). The lichenase (1,3;1,4-endo-β-D-glucan endohydrolase) and endo-β-xylanase assays were performed using K-MBG4 and K-XylX6 commercial kits, respectively (Megazyme, Bray, Ireland) according to the instructions of the manufacturer. The experiment was performed at least in three biological replicates for each type of activity.

β-D-Galactosidase and β-D-glucosidase activities were detected on cross sections of the primary root of maize seedlings using the resorufin-β-D-galactopyranose (Res-βDGal*p*) and resorufin-β-D-glucopyranose (Res-βDGlc*p*) substrates (Merck, Kenilworth, NJ, United States). The substrates were resolved in dimethyl sulfoxide and then diluted with 0.2 M phosphate-buffered saline (PBS, pH 7.4) to 100 μM concentration. Ten microliters of the substrate were added to each cross section. Twenty-five and 50 μm-thick sections were made on a vibratome (Leica Biosystems, Wetzlar, Germany) in the middle part of each analyzed maize root zone (Figure 2). Enzyme activity was detected by red fluorescence on a Leica DM1000 epifluorescence microscope (Leica Biosystems, Wetzlar, Germany) fitted with a mercury lamp and filter cube with excitation at 540-580 nm and extinction at 608-683 nm. Exposure time was maintained constant. The sections were observed after 10 min of reaction. The control sections were heated to 90°C for 5 min in a water drop and then treated with substrates. Images were obtained using a digital camera. The experiment was performed in three biological replicates.

### RNA Extraction and Sequencing

In addition to the previously published data (Kozlova et al., 2020, BioProject: PRJNA639682), twenty more individual libraries of mRNAs were obtained from the same five zones of the maize primary root in four biological replicates were analyzed using an Illumina sequencing technology. Root segments of ca. 30 plants were collected in plastic tubes with liquid nitrogen and stored in a freezer at −80°C until mRNA extraction. Total RNA was isolated from the plant samples using the TRIzol-based extraction method in combination with RNeasy Plant Mini Kit (Qiagen, Hilden, Germany) according to the instructions of the manufacturer. Residual DNA was removed by treating the samples with a Turbo DNA-free kit (Ambion, Austin, TX, United States). The quantity and quality of RNA were controlled using NanoPhotometer NP-80 Touch (Implen, Munich, Germany) and by electrophoresis in 1% (w/w) agarose gel. RNA integrity test (all samples had a RIN value higher than 7), cDNA library construction, and sequencing were performed by Novogene Company Limited (Cambridge, United Kingdom^1^). Data in the form of raw reads and sample preparation descriptions were added to BioProject number PRJNA639682^2^ in the Sequence Read Archive (SRA).

### Processing and Analysis of RNA-seq Data

Low-quality reads, reads containing adapters, and contaminating sequences were removed with the BBDuk utility of the BBToolsv38.73^3^ tool to get clean read data. Paired-end clean reads for each sample were mapped onto the maize genome version B73 RefGen_v4 downloaded from Gramene^4^ using HISAT2 v2.1.(Kim et al., 2015). The StringTie v2.0. software (Pertea et al., 2016) was employed to count the number of reads mapped to each gene. Normalized TGR (total gene read) values were used as input for differential expression analyses using R package DESeq2 v1.30.1 after normalization of the combined dataset (previously generated by us (Kozlova et al., 2020, BioProject: PRJNA639682) and in this study) in a DESeq2’s statistical model (Love et al., 2014)^5^. A gene was considered as expressed if the average normalized TGR in at least one sample was > = 16 according to the recommendations of the sequencing quality control project (Su et al., 2014). The resulting dataset consisted of 28,888 genes. The transcriptomic data were combined with the results of the proteomic study performed on maize primary root by C. Marcon et al. (2015). Details of the maize root sampling in both studies can be found in Figure 2.

### Cluster and Co-Expression Analyses

A set of expressed genes encoding maize GHs in the form of rlog-transformed values (the regularized-logarithm transformation of count data; Love et al., 2014) was used as input for R packages (hclust, heatmap.2, gplots, dendextend) to generate clusters. The normalized expression matrix (TGR > = 16 in at least one sample) was used to generate the targeted co-expression networks by the Comparative Co-Expression Network Construction and Visualization tool (CoExpNetViz) using the Pearson correlation coefficient and “bait” genes that are supposed to be involved in the same biological process or pathway (Tzfadia et al., 2016). The following genes were used as baits: six genes encoding putative maize cellulose synthases of the primary cell wall (*Zm00001d019317*, *Zm00001d037636*, *Zm00001d005250*, *Zm00001d019149*, *Zm00001d009795*, and *Zm00001d005478*); four genes encoding putative maize cellulose synthases of the secondary cell wall (*Zm00001d043477*, *Zm00001d032776*, *Zm00001d020531*, and *Zm00001d005775*); maize homolog of main XyG backbone synthase of rice (*Zm00001d04933*) (Penning et al., 2019; Kozlova et al., 2020). The co-expression networks were visualized using Cytoscape version 3.8.1. (Shannon et al., 2003)^6^.

### Search for Genes Encoding Maize Polysaccharide Hydrolases and Phylogenetic Analysis

Putative GHs were recognized in the maize B73 genome (RefGen_AGPv4) by the presence of characteristic Pfam domains in the predicted full-length protein sequences. The protein sequences of the maize genome were downloaded from the Ensembl Plants database (release 47) (Bolser et al., 2016)^7^. Domain search was performed in the HMMer3.3 software using the following domain profiles: PF00232 – GH1; PF01915, PF00933 – GH3; PF00759 – GH9; PF00331 – GH10; PF00722 – GH16; PF00332 – GH17; PF00295 – GH28; PF01301 – GH35; PF06964 – GH51, PF14498 – GH95. The domain profiles were downloaded from Pfam32.0 (El-Gebali et al., 2019)^8^. Sequences with *E*-values higher than 1 × 10^–6^ were discarded. Protein sequences with high *E*-values were additionally checked in the InterProScan tool implemented in the InterPro database (Mitchell et al., 2019)^9^ to confirm the presence of GH domains. Signal peptide presence was predicted with the SignalP tool (Armenteros et al., 2019)^10^.

The obtained maize transcripts of GHs were aligned between each other within each GH family using ClustalW (Madeira et al., 2019)^11^. The longest transcripts encoding the sequence with catalytic acid/base residues (revealed by literature data) were used for further phylogenetic analysis. Genes with only one transcript encoding the sequence shorter than 150 amino acids were recognized as pseudogenes.

The protein sequences of *Arabidopsis thaliana* GHs were recognized by Pfam domain name or gene name search and downloaded from the Phytozome v12.1 database (Goodstein et al., 2012)^12^ or from the Uniprot database (release 2020_05) (Consortium, 2019)^13^. The protein sequences of other plant genes were obtained from the Uniprot database and from the NCBI Protein database (GenPept) (Benson et al., 2012)^14^. Phylogenetic trees were built as described previously (Kozlova et al., 2020) using the IQ-TREE software (Nguyen et al., 2015). In the case when shortened gene names are used in the trees, corresponding locus IDs can be found in Supplementary Table 1.

## Results

Cluster analysis of gene expression performed on all investigated GHs revealed four major clusters (Figure 2). Cluster I was characterized by the highest levels of expression in root cap and meristem with further decrease. A similar expression pattern in maize root was shown by glycosyltransferases (GTs) putatively involved in the synthesis of XyG (Kozlova et al., 2020). Cluster II was composed of genes whose transcript abundance peaked in the zone of early elongation and was minimal in both root cap and late elongation zone. Similar dynamics of transcript levels in maize root were demonstrated by GTs related to homogalacturonan (HG) biosynthesis (Kozlova et al., 2020). Cluster III contained genes whose expression was highest in the stage of active elongation with a subsequent decrease in the late elongation zone. Such character of expression was typical for GTs involved in the synthesis of primary cell wall cellulose, MLGs, rhamnogalacturonans (RGs), and GAXs (Kozlova et al., 2020). These polysaccharides are abundant in the primary cell wall of grasses. Cluster IV was composed of genes whose transcription was low in the early elongation zone but increased in the active elongation and, especially, late elongation zones.

### Expression of Genes Encoding Exo-β-Glucosidases and Endo-β-Glucanases

The glucose-containing polysaccharides of cell walls include only β-linked glucose residues. Thus, we have focused on exo-β-glucosidases (EC 3.2.1.21), which act on terminal glucose, and endo-β-glucanases (EC 3.2.1.4), which are supposed to cut β-glucan backbones. According to the CAZy database (Lombard et al., 2014)^15^, plant β-glucosidases occur in the GH1 and GH3 families.

**FIGURE 3:**
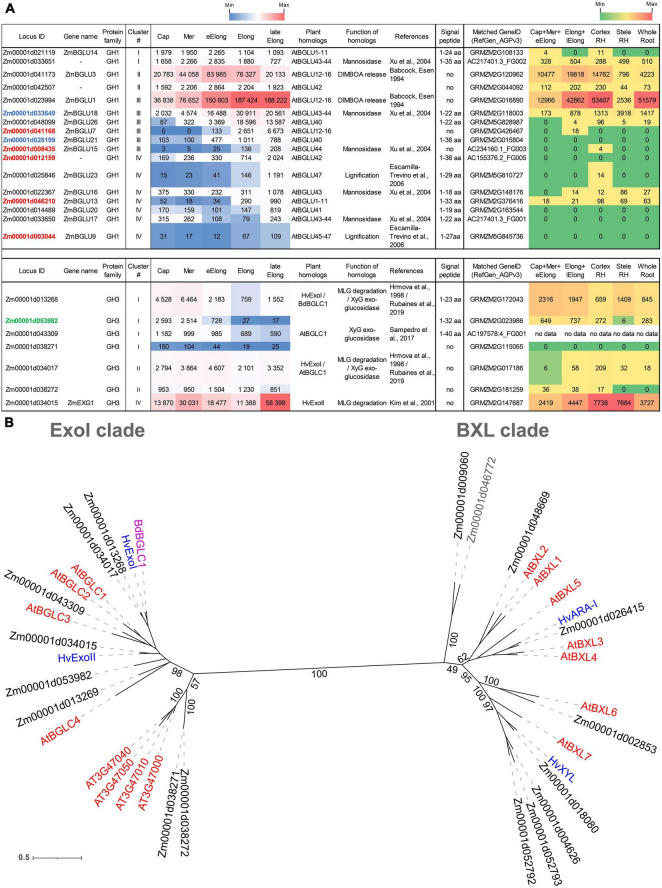
Expression of maize genes encoding putative GH1 and GH3 β-D-glucosidases in maize roots and phylogenetic analysis of plant GH3 family members. **(A)** The level of transcripts (TGR, red-blue heat map) and abundance of corresponding protein (averaged and normalized total spectral counts (Marcon et al., 2015, red-green heat map) of genes encoding putative β-D-glucosidases of GH1 and GH3 protein families in analyzed zones of maize roots. Heat map color-coding was applied to each protein family separately. Genes with expression values below 100 in all the studied samples are not shown. TGR values are sorted from maximum to minimum within each cluster. Maize genes that co-expressed with primary and secondary cell wall cellulose-synthases, and with the XyG backbone synthase are given in blue, red, and green, respectively. Maize GH1 gene names are given according to Gómez-Anduro et al. (2011). ZmEXG1 is named after Kim et al. (2000). Cap, root cap; Mer, meristem; eElong, early elongation zone; Elong, zone of active elongation; lateElong, zone of late elongation before root hair initiation; RH, root hair zone; aa, amino acids; no data, no corresponding peptides were found by Marcon et al. (2015) proteomic analysis. **(B)** The unrooted maximum likelihood phylogenetic tree of plant GH3 members. Maize expressed genes are given in black, and unexpressed (TGR values lower than 16 in all the analyzed root samples) in gray, *Arabidopsis thaliana* genes in red, and barley in blue (only genes encoding enzymes with characterized enzymatic activity are shown (Hrmova et al., 1996; Hrmova and Fincher, 1997; Lee et al., 2003), *Brachypodium distachyon BdBGLC1* (*Bd1g08550*) (Rubianes et al., 2019) is given in purple. The *A. thaliana* gene names follow Minic et al. (2004) for the BXL clade and follow Sampedro et al. (2017) for the ExoI clade. Numbers indicate ultrafast bootstrap support values for some branches.

We identified 29 genes encoding proteins of the GH1 family in the maize genome of the fourth assembly (RefGen_B73 AGPv4). All of them possess catalytic nucleophiles and acid/base glutamic acids according to multiple alignments (data not shown). Twenty-two out of the 29 revealed that GH1 genes were expressed in the maize primary root (Figure 3A).

The genes encoding GH1 members are distributed over all four clusters. Cluster III (maximum of TGR values in the zone of active elongation) included *ZmBGLU1* that had extremely high TGR values and the highest content of corresponding protein among all the maize GT and GH genes studied (Kozlova et al., 2020; Figure 3A). The ZmBGLU1 enzyme is cytosolic and participates in anti-insect defense by releasing aromatic compound DIMBOA from its glucoside form (Babcock and Esen, 1994). The *ZmBGLU3* gene, a homolog of *ZmBGLU1* also expressed at a very high level, however, joined cluster II (Figure 3A). The other member of cluster III with a high level of transcripts and high abundance of corresponding protein was *ZmBGLU18*. The ZmBGLU18 homologs in *Arabidopsis* and tomato, AtBGLU43-44 (Xu et al., 2004) and LeMside1 (Mo and Bewley, 2002), were proven to serve as β-mannosidases (EC 3.2.1.25). In the phylogenetic dendrogram of the GH1 protein family, several other maize genes besides *ZmBGLU18* shear the clade with *AtBGLU43-44* (Supplementary Figure 1). They belong to clusters I, III, and IV, and some of them are characterized by considerable TGR values and protein content (Figure 3A). Thus, highly expressed in maize root isoforms of GH1 proteins are either cytosolic and defense-related or putative mannosidases, and barely contribute to MLG metabolism during growth.

Another protein family that contains putative exo-β-D-glucosidases is GH3. In the maize genome, we identified 17 maize genes encoding GH3 proteins by the simultaneous presence of PF00933 (Glyco_hydro_3, GH3 N-terminal domain) and PF01915 (Glyco_hydro_3_C, GH3 C-terminal domain) Pfam domains in their predicted protein sequences. The phylogenetic tree of GH3 diverges into two distant clades (Figure 3B). The ExoI clade was named after the characterized barley β-glucosidase HvExoI (Hrmova et al., 1998). Besides this barley gene, the ExoI clade contains eight maize genes including *ZmEXG1*, whose product was characterized as β-glucosidase in maize coleoptiles (Kim et al., 2000), and *AtBGLC1-4* encoding β-glucosidases of *A. thaliana* attacking XyGs oligosaccharides (Sampedro et al., 2017; Figure 3B). The second clade, BXL, is formed by nine maize homologs of *A. thaliana AtBXL1-7* encoding β-xylosidases/arabinofuranosidases (Minic et al., 2004; Figure 3B).

Among all genes encoding GH3 members, *ZmEXG1* was characterized by the highest levels of mRNA in all the maize root zones studied (Figure 3A). The expression pattern of *ZmEXG1* demonstrated two peaks: one in the meristem zone with a subsequent decrease during initiation and active elongation growth, and then a dramatic increase in the late elongation stage (Figure 3A). The protein content of ZmEXG1 was found to be very high in the elongating part of the root (Marcon et al., 2015). Two maize homologs of *HvExoI* (*Zm00001d034017* and *Zm00001d013268*) displayed similar two-peak dynamics of transcript abundance along the maize root (Figure 3A). The amount of protein for the *Zm00001d013268* gene was at a very high level in maize root (Marcon et al., 2015).

Another GH3 member characterized by a considerable amount of detected proteins was *Zm00001d053982*. It belonged to cluster I, with the peak of TGR values in the root cap and meristem zone with a gradual decrease in subsequent zones. *Zm00001d053982* co-expressed with the *Zm00001d049336* gene, which encodes the putative maize XyG backbone synthase. The *Zm00001d049336* gene is a close homolog (Kozlova et al., 2020) of rice *OsCslC3* (Liu et al., 2015).

Besides exo-glucosidases, glucans can be utilized *in planta* by endo-glucanases, which act on non-terminal bonds releasing oligo- or polymer products but not monosaccharides. Plant endo-glucanases are thought to belong to the GH9 and GH17 families.

We identified 27 genes encoding GH9 family proteins in the maize genome by the presence of the PF00759 (Glyco_hydro_9) domain in their predicted amino acid sequences. The gene *Zm00001d002943* is annotated as methyltransferase (Methyltransf_29). However, three out of forty transcripts of this gene encoded sequences with the Glyco_hydro_9 domain instead of the Methyltransf_29 domain. Thus, in our study, it was included in the GH9 family (Supplementary Figure 2). Twenty-three out of the 27 identified GH9 genes were expressed in maize root with TGR values higher than 16 in at least one root zone (Supplementary Figure 2A). The highest expression level was displayed by maize genes (*Zm00001d002943*, *Zm00001d026712*, *Zm00001d021304*, *Zm00001d033917*, and *Zm00001d013319*) homologous to *KORRIGAN1-3* (*KOR1-3*) of *A. thaliana* (Supplementary Figure 2). KORRIGANs are parts of the cellulose synthase complex in many plants starting from the earliest taxa (Lampugnani et al., 2019). Maize homologs of KORRIGANs belonged to the third cluster demonstrating an increasing level of transcript abundance from root cap to the active or late elongation zone. Cluster III also contained maize genes that are homologous to *AtEG11 (At2g32990)* of *A. thaliana* (*Zm00001d044744*, *Zm00001d017978*, *Zm00001d015292*, *Zm00001d020371*, and *Zm00001d051814*) (Supplementary Figure 2). Three of them were co-expressed with secondary cell wall cellulose synthases (Supplementary Figure 2A). Corresponding proteins for many of these genes were found in the proteomics study by Marcon et al. (2015) mainly in the root hair zone (Supplementary Figure 2).

Maize homologs of *AtCEL1*, the gene whose protein product was found to be important for cell wall relaxation during growth in *A. thaliana* (Tsabary et al., 2003), and maize homologs of the related *AtCEL2,3,5* genes transcribed at a significant level only in the root cap falling into cluster I (Supplementary Figure 2). Their proteins were detected mainly in the root segment combining root cap, meristem, and early elongation zone (Marcon et al., 2015). This well agrees with the root cap-specific expression of *AtCEL5* and its redundancy in the *Arabidopsis* genome reported by Del Campillo et al. (2004). Maize homologs of *OsGLU1*, a rice gene encoding an enzyme able to cleave β-1,4-glucans and graminaceous hemicelluloses (Yoshida and Komae, 2006), transcribed at a very low level, and no protein products for them were found along the maize root (Supplementary Figure 2). Cluster IV was represented by genes having low levels of transcript and protein abundance (Supplementary Figure 2). It seems feasible that maize homologs of KORRIGANs, AtEG11, and AtCEL1-5 participate in cellulose biosynthesis in different cell types.

Maize cell walls are capable of autolysis during growth. Hatfield and Nevins purified endoglucanase from maize coleoptiles and characterized it as an enzyme able to cleave MLG, carboxymethylcellulose, and XyG (Hatfield and Nevins, 1986, 1987). The sequence of more than three β-1,4-linked glucose is required for recognition and hydrolysis by this endoglucanase (Hatfield and Nevins, 1987). A BLAST search using the N-terminal amino acid sequence of endoglucanase as query (Inouhe et al., 1999) revealed two corresponding maize *Zm00001d050196* and *Zm00001d050198* genes. Transcript levels of these genes in maize roots were high and significantly increased during elongation growth (Supplementary Figure 2). *Zm00001d050198* was co-expressed with primary cell wall-related cellulose-synthases and MLG-synthases, and corresponding proteins were detected at a very high level (Marcon et al., 2015; Supplementary Figure 2). However, protein sequences encoded by the *Zm00001d050196* and *Zm00001d050198* genes do not possess any known glycoside hydrolase domains. They are annotated as alpha/beta hydrolases and possess an Abhydrolase_5 (PF12695) Pfam domain.

In the maize genome, 58 genes encoding proteins that possess a Glyco_hydro_17 (PF00332) Pfam domain were identified. The list was reduced to 51 genes by the presence of catalytic glutamic acids that were established by multiple alignments with protein sequences of barley HvEII and HvGII endo-hydrolases. Their crystal structures and catalytic mechanisms were resolved (Varghese et al., 1994; Müller et al., 1998). Forty genes of GH17 were transcribed with TGR values higher than 16 in at least one analyzed root zone (Supplementary Figure 3).

In maize root, all the four main clusters of gene expression were represented by genes encoding GH17 proteins. In cluster I, which joins genes with the peak of TGR values in the root cap and meristem, for almost all genes, corresponding peptides were detected (Supplementary Figure 3). The two maize homologs (*Zm00001d028243* and *Zm00001d048055*) of *Arabidopsis PdBG3* also joined cluster I. *PdBG3* was shown to be involved in lateral root formation by the control of callose accumulation (Benitez-Alfonso et al., 2013). Cluster II that included genes with a peak of TGR values in the early elongation zone was highly populated by the GH17 genes. No homologs of any characterized plant enzymes were among them. Clusters III and IV included many genes that were co-expressed with secondary cell wall cellulose-synthases. The corresponding proteins were found predominantly in the stele of the root hair zone (Marcon et al., 2015; Supplementary Figure 3). Cluster IV also joined the maize homolog (*Zm00001d038049*) of barley *HvEI-HvEII* (Supplementary Figure 3). Barley *HvEI-HvEII* enzymes are 1,3;1,4-β-D-glucan endohydrolases (Varghese et al., 1994). The *Zm00001d038049* gene matches the maize *GRMZM2G137535* gene, which was demonstrated to be an MLG-degrading enzyme in maize leaves (Kraemer et al., 2021). The protein content of *Zm00001d038049* in roots was negligible (Supplementary Figure 3).

### Expression of Genes Encoding β-D-Galactosidases

**FIGURE 4:**
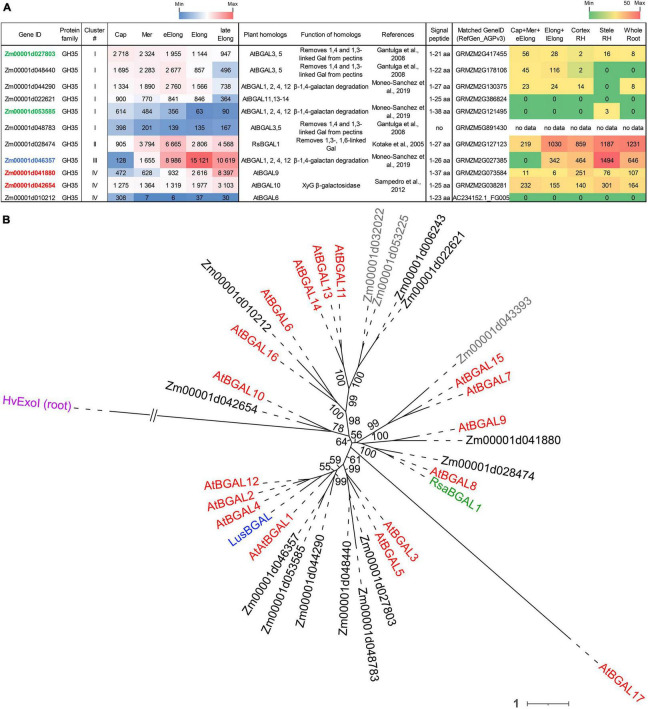
Expression of maize genes encoding putative GH35 β-D-galactosidases in maize roots and phylogenetic analysis of plant GH35 family. **(A)** The level of transcripts (TGR, red-blue heat map) and abundance of corresponding proteins (averaged and normalized total spectral counts (Marcon et al., 2015), red-green heat map) of genes encoding putative β-D-galactosidases of the GH35 protein family in various zones of maize roots. Genes with expression values below 100 in all the studied samples are not shown. TGR values are sorted from maximum to minimum within each cluster. Maize genes co-expressed with primary and secondary cell wall cellulose-synthases, and with the XyG backbone synthase are given in blue, red, and green, respectively. XyG, xyloglucan; Gal, galactose; aa, amino acids; Cap, root cap; Mer, meristem; eElong, early elongation zone; Elong, zone of active elongation; lateElong, zone of late elongation before root hair initiation; RH, root hair zone; no data, no corresponding peptides were found by Marcon et al. (2015) proteomic analysis. **(B)** Unrooted maximum likelihood phylogenetic tree of GH35 protein family members. Maize expressed genes are given in black, and unexpressed (TGR values lower than 16 in all the analyzed root samples) in gray, *Arabidopsis thaliana* genes in red, flax *LusBGAL* (Roach et al., 2011) in blue, and radish *RsBGAL1* (Kotake et al., 2005) in green. *Arabidopsis* gene names follow Chandrasekar and van der Hoorn (2016). *HvExoI* was used as outgroup, and branch length was shortened. Numbers indicate ultrafast bootstrap support values for some branches.

β-D-Galactosidases (EC 3.2.1.23) are enzymes that cleave off terminal β-D-galactose. In higher plants, β-D-galactosidases can act on the side chains of XyG, pectins, and other galactose-containing substrates. Plant β-D-galactosidases belong exclusively to the GH35 protein family according to the CAZy database and possess the Glyco_hydro_35 (PF01301) domain. Two glutamic acids in conserved motifs Q-x-E-N-**E**- and W-T-**E**-x-W serve as the proton donor and nucleophile of GH35, respectively (Chandrasekar and van der Hoorn, 2016), so sequences lacking one of these motifs were excluded from further analysis. We identified 14 putative maize β-D-galactosidases, and 11 of them were transcribed in the maize root samples with TGR values higher than 100 in at least one root zone (Figure 4).

The expression of about half of the GH35 maize genes corresponded to cluster I with the highest TGR values in the root cap and meristem zones of maize roots (Figure 4A). All of them join the same clade as *A. thaliana* pectin-related β-galactosidases *AtBGAL1* and *AtBGAL5* (Gantulga et al., 2008) in the phylogenetic tree except for *Zm00001d022621* (Figure 4B). However, these genes were characterized by moderate protein content.

The sole maize β-galactosidase (*Zm00001d028474*) resides in cluster II. It is a homolog for *AtBGAL8* and radish *RsBGAL1*. The latter was characterized as β-galactosidase with specificity to 1,3- and 1,6-linked galactose, but not to 1,4-linked galactose (Kotake et al., 2005). Interestingly, the transcript abundance of this maize gene sharply decreased in the active elongation zone in a way similar to *ZmEXG1* expression (Figure 3A). Despite moderate gene expression, the protein products of *Zm00001d028474* accumulated in the elongation zone at a very high level (Figure 4A). The *Zm00001d046357* gene demonstrated the highest TGR values among GH35 members in maize root with the peak of expression in the active elongation zone. *Zm00001d046357* joined cluster III and is one of the closest maize homologs for *Arabidopsis AtBGAL1,2,4,12* and flax *LusBGAL* (Figure 4B). AtBGAL1, 2, 3, 5, and LusBGAL are involved in the trimming of β-1,4-galactan side chains of RGs-I in *Arabidopsis* and flax, respectively (Gantulga et al., 2008; Roach et al., 2011; Moneo-Sánchez et al., 2018). *Zm00001d046357* joined the co-expression network based on primary cell wall cellulose synthases that also included *ZmGALS1*, the predicted maize β-1,4-galactan synthase (Kozlova et al., 2020).

The cluster IV joined *Zm00001d041880* and *Zm00001d042654* homologs of *AtBGAL9* and *AtBGAL10*, respectively. They displayed considerable levels of transcripts and proteins along the maize root (Figure 4) and co-expressed with secondary cell wall-related cellulose synthases. AtGAL10 is the main β-galactosidase of *A. thaliana* with specificity against XyG. This gene is highly expressed in elongating tissues. The insertional mutation of *AtGAL10* resulted in an unusual pattern of side chain distribution along the XyG molecule and growth defects (Sampedro et al., 2012).

### Expression of Genes Encoding Pectinases

Plant pectin-related GHs include exo- (EC 3.2.1.67) and endo-polygalacturonases (EC 3.2.1.15) belonging to the GH28 family. The maize genome contains 53 genes encoding GH28 members, although only 27 of them had TGR values over 16 in at least one studied root zone (Supplementary Figure 4). All maize genes whose transcript and protein abundance in maize root varied significantly during growth belonged to one divergent clade of the GH28 phylogenetic tree (Supplementary Figure 4B). In *Arabidopsis*, this clade was defined by Cao (2012) as the group V of polygalacturonases. This clade still has no characterized representatives. Genes encoding maize genes homologous to studied plant enzymes had relatively low transcript levels, and no proteins were detected (Supplementary Figure 4).

### Expression of Genes Encoding Enzymes Degrading Xylose- and Arabinose-Containing Substrates

**FIGURE 5:**
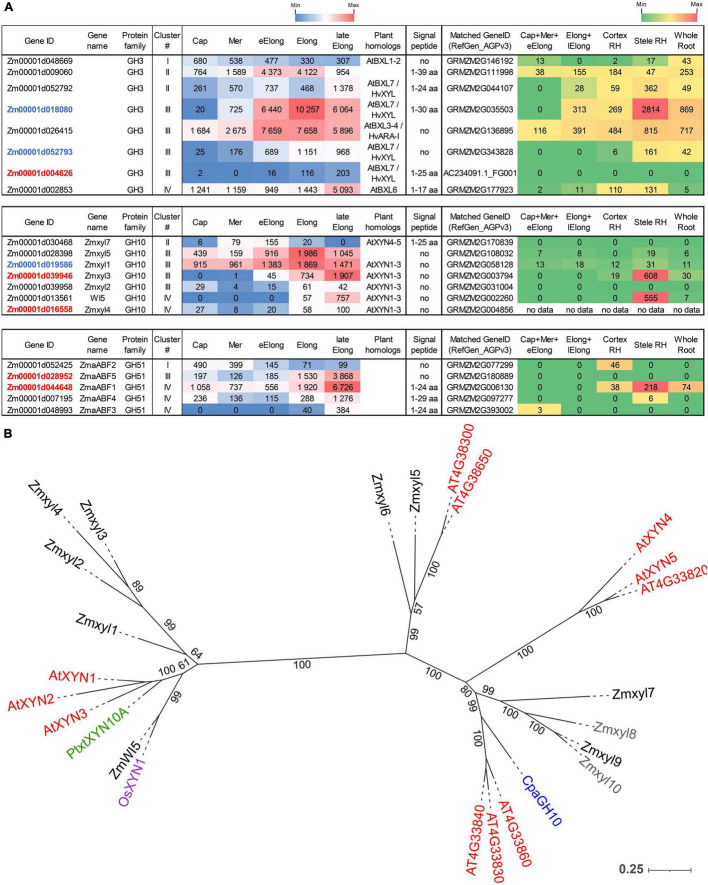
Expression of maize genes encoding putative β-D-xylosidases, xylanases, and α-L-arabinofuranosidases of the GH3, GH10, and GH51 families in maize roots and phylogenetic analysis of plant GH10 family. **(A)** The level of transcripts (TGR, red-blue heat map) and abundance of corresponding proteins (averaged and normalized total spectral counts (Marcon et al., 2015), red-green heat map) of genes encoding putative β-D-xylosidases, xylanases, and α-L-arabinofuranosidases in various zones of maize roots. Genes with expression values below 100 in all the studied samples are not shown. TGR values are sorted from maximum to minimum within each cluster. Maize genes co-expressed with primary and secondary cell wall cellulose-synthases are given in blue and red, respectively. Maize gene names for GH10 follow Hu et al. (2020). Maize gene names for GH51 follow Kozlova et al. (2015). Cap, root cap; Mer, meristem; eElong, early elongation zone; Elong, zone of active elongation; lateElong, zone of late elongation before root hair initiation; RH, root hair zone; aa, amino acids; no data, no corresponding peptides were found in any of the studied samples. **(B)** Unrooted maximum likelihood phylogenetic dendrogram of plant GH10 family members. Maize expressed genes are given in black, and unexpressed (TGR values lower than 16 in all the analyzed root samples) in gray, *Arabidopsis thaliana* in red, poplar (Derba-Maceluch et al., 2015), rice (Tu et al., 2020), and papaya (Johnston et al., 2013) genes are given in green, pink, and purple, respectively. *Arabidopsis thaliana* gene names follow Suzuki et al. (2002), and maize gene names follow Hu et al. (2020). Numbers indicate ultrafast bootstrap support values.

Plant β-xylosidases (EC 3.2.1.37) belong to the GH3 family. They occupy one of the two clades of GH3, which is designated as the BXL clade (Figure 3B). Nine maize genes occupy the BXL clade, and eight of them were expressed in maize root (Figure 5). Besides maize genes, the BXL clade includes *Arabidopsis* genes encoding β-xylosidases and bifunctional arabinofuranosidase/β-xylosidase *AtBXL1-7* (Minic et al., 2004), and barley *HvARA-I* and *HvXYL* (Lee et al., 2003).

The highest transcript and protein levels among maize GH3 BXL clade members were shown by the *Zm00001d018080* and *Zm00001d026415* genes (Figure 5A). According to the phylogenetic dendrogram of GH3, the *Zm00001d018080* and *Zm00001d026415* genes are homologs of *HvXYL* and *HvARA-I*, respectively (Figure 3B). HvXYL and HvARA-I are β-xylosidase and bifunctional α-L-arabinofuranosidase/β-D-xylosidase, respectively, that were purified from barley seedlings (Lee et al., 2003). Their maize homologs had peak TGR values in the active elongation stage and significant protein content in maize root (Marcon et al., 2015; Figure 5A). Relatively low levels of transcript and protein abundances were displayed by maize BXL clade members representing clusters I and IV (Figure 5A). Cluster II (TGR values are highest in the early elongation zone) included two other members of this clade. One of them (*Zm00001d052792*) is also a homolog of *HvXYL*, while the other (*Zm00001d009060*) is located in the remote branch of the GH3 phylogenetic tree rather distant from any of its characterized members (Figure 3B).

Enzymes called xylanases (EC 3.2.1.8) cleave the backbone of xylan chains and act as endo-hydrolases. Seventeen GH families contain endo-xylanases according to the CAZy database; however, plant endo-xylanases reside only in the GH10 protein family (Hu et al., 2020). The phylogenetic tree of the plant GH10 family splits into two clades (Figure 5B). One clade includes the majority of plant xylanases characterized to date, *Arabidopsis* AtXYN-1, *Populus* PtxtXYN10A, maize WI5, and rice OsXYN1 (Figure 5B). For all of them, roles in secondary cell wall formation and vascular system differentiation have been shown (Suzuki et al., 2002; Derba-Maceluch et al., 2015; Hu et al., 2020; Tu et al., 2020). The other clade of GH10 has only two characterized members. One of them, ZmXYL, was described as the most abundant enzyme on the surface of maize pollen grains (Bih et al., 1999). This enzyme operates as xylanase, which facilitates pollen tube penetration through the silk by means of xylan degradation (Suen and Huang, 2007). We designated it as *Zmxyl7* following Hu et al. (2020). Another characterized member of this clade is papaya *CpaEXY1* (*CpaGH10*), the xylanase that acts during fruit softening (Johnston et al., 2013; Figure 5B).

Eleven maize genes encoding putative xylanases were identified in the maize genome by Hu et al. (2020). Nine of them were transcribed in primary maize root (Figure 5A). The *Zmxyl1* and *Zmxyl5* genes displayed the highest transcript levels in the elongation zone, but their protein content was low (Figure 5A). *Zmxyl3* had its highest TGR value in the late elongation zone similar to *ZmWI5*. The corresponding proteins for both these genes were found exclusively in the stele of the root hair zone (Marcon et al., 2015; Figure 5A).

Plant α-L-arabinofuranosidases (EC 3.2.1.55) were classified into the GH3 and GH51 families (CAZy database). Putative maize GH51 α-L-arabinofuranosidases were identified by the presence of characteristic PF06964 domain and named following our previous study (Kozlova et al., 2015). Five out of six predicted GH51 genes were transcribed in maize root. *ZmaABF5* and *ZmaABF1* were expressed at high levels in the late elongation zone and co-expressed with maize secondary cell wall cellulose synthases. Proteins of α-L-arabinofuranosidases were detected only in the root hair zone of maize root (Figure 5A).

### Expression of Genes Encoding Xyloglucan Endotransglycosylases/Hydrolases

Xyloglucan (XyGs) can be modified *in planta* by xyloglucan endotransglucosylases/hydrolases (XTHs) belonging to the GH16 protein family. This group is composed of XyG endohydrolases (EC 3.2.1.151, XEHs) and XyG endotransglycosylases (EC 2.4.1.207, XETs). XEHs cleave the backbone of XyGs as usual β-1,4-endoglucanases, releasing two parts of an initial polymer as individual molecules. In the case of XETs, the first step of catalysis is the same but the non-reducing terminus of a cleaved XyG is retained in the catalytic site and serves as donor substrate for further stage. In the second step, the XET enzyme transfers the bound portion of XyG into another XyG molecule (acceptor). This type of reaction is called homo-transglycosylation. When either a donor or an acceptor substrate is presented by a polysaccharide other than XyG, the reaction is described as hetero-transglycosylation (Stratilova et al., 2020).

**FIGURE 6:**
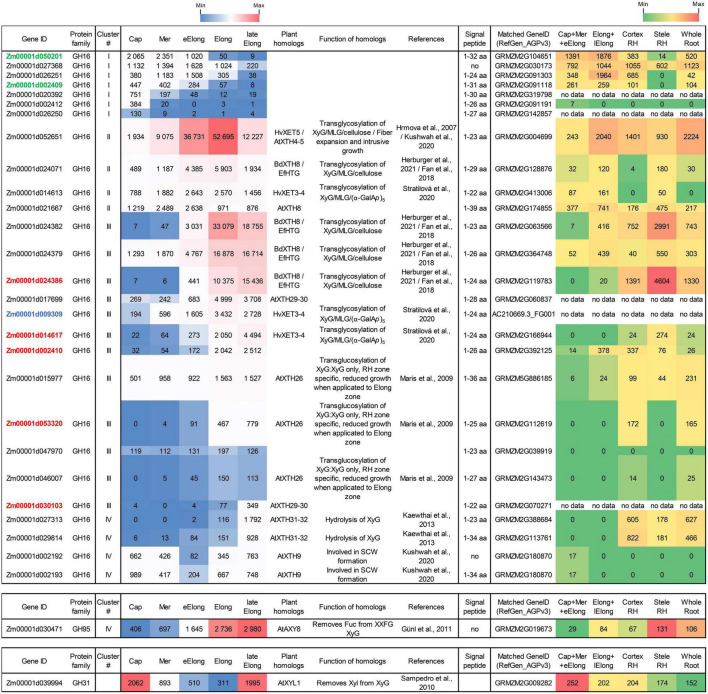
Expression of maize genes encoding putative xyloglucan endotransglucosylases/hydrolases (XTHs) of GH16 in maize roots. The level of transcripts (TGR, red-blue heat map) and abundance of corresponding proteins (averaged and normalized total spectral counts (Marcon et al., 2015), red-green heat map) of genes encoding putative XyG endotransglycosylases/hydrolases of the GH16 protein family in various zones of maize roots. Genes with expression values below 100 in all the studied samples are not shown. TGR values are sorted from maximum to minimum within each cluster. Maize genes co-expressed with primary and secondary cell wall cellulose-synthases, and the XyG backbone synthase are labeled in blue, red, and green, respectively. XyG, xyloglucan; MLG, mixed-linkage glucan; SCW, secondary cell wall, Cap, root cap; Mer, meristem; eElong, early elongation zone; Elong, zone of active elongation; lateElong, zone of late elongation before root hair initiation; RH, root hair zone; no data, no corresponding peptides were found by Marcon et al. (2015) proteomics analysis.

**FIGURE 7:**
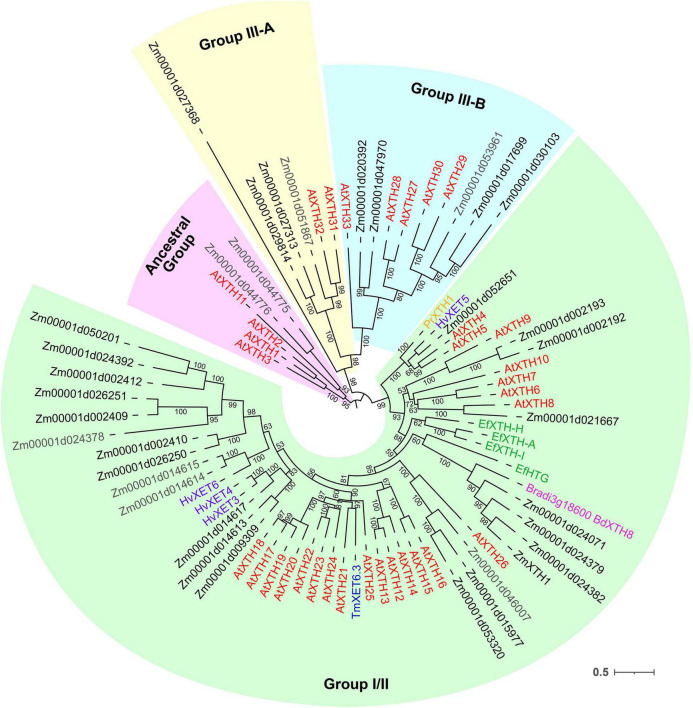
Unrooted maximum likelihood phylogenetic analysis of the plant GH16 family. Maize genes are given in black font for expressed genes and gray for those having TGR values lower than 16 in all the analyzed root samples, and *Arabidopsis thaliana* genes are given in red. Characterized XTHs of barley (Hrmova et al., 2007, 2009), *Equisetum* (Herburger et al., 2021), nasturtium (Crombie et al., 1998), and *Brachypodium* (Fan et al., 2018) are shown in purple, green, blue, and magenta, respectively. Numbers indicate ultrafast bootstrap support values for some branches.

Xyloglucan endotransglucosylases/hydrolases (XTHs) were recognized in the maize genome by the presence of the PF00722 domain and conserved motif E-x-D-x-E. A total of 36 putative XTH genes were identified in the maize genome, and 31 of them were expressed in maize primary root (Figures 6, 7).

Maize genes that demonstrate peak of transcription in the root cap and meristem joined cluster I. Most of them represented the *Poaceae*-specific subclade in the plant GH16 phylogenetic tree together with barley hetero-transglycosylases *HvXET3, 4*, and *6* (Hrmova et al., 2009; Stratilova et al., 2020). Despite moderate TGR values of cluster I members, the content of corresponding proteins was extremely high in the elongating part of the root (Marcon et al., 2015; Figure 6). Two members of cluster I (*Zm00001d050201* and *Zm00001d002409*) were co-expressed with the putative XyG backbone synthase of maize (Figure 6).

Cluster II contained the *Zm00001d052651*gene that displayed the highest TGR values among all the maize XTHs (Figure 6). The *Zm00001d052651* gene is the sole homolog to *Arabidopsis AtXTH4*, and *5*, and barley *HvXET5* (Figure 7). AtXTH4 is involved in cell wall remodeling during fiber expansion and intrusive growth (Kushwah et al., 2020) in *Arabidopsis*. HvXET5 was described as a hetero-transglycosylase, which mediates XyGs tailoring to MLG and cellulose (Hrmova et al., 2007) in barley. The protein abundance of *Zm00001d052651* increased 10 times between the maize root tip and root hair zone (Figure 6). Two more putative hetero-XETs of maize, homologous to *BdXTH8* of *Brachypodium* and *HvXET3,4* of barley, belonged to cluster II (Figure 6). BdXTH8 connects XyG and MLG (Fan et al., 2018), and HvXET3-4 are able to join XyG and HG pentasaccharides (Stratilova et al., 2020). Corresponding proteins were abundant in the elongating part of the maize root (Figure 6).

The highest TGR values and protein content in cluster III (expression is maximum at active elongation) were also displayed by maize homologs of *Brachypodium BdXTH8* and barley *HvXET3-4* (Figure 6). The transcript and protein abundance of putative maize hetero-transglycosylases of clusters II and III indicates the importance of reactions of this type for elongation growth initiation and realization. Putative maize homo-transglycosylases [homologs of *AtXTH26* that were characterized by Maris et al. (2009)] were also present in cluster III. Their TGR values and protein levels were lower than those of hetero-transglycosylases of the same cluster (Figure 6). The highest transcription levels in cluster IV (late elongation zone-specific) were demonstrated by maize homologs of *AtXTH31* and *32* (Figure 7). These genes encode XEHs in *Arabidopsis* (Kaewthai et al., 2013). Proteins of their maize homologs appeared only in the root hair zone of maize primary root (Figure 6).

### Glycoside Hydrolase Activities Along Maize Root

**FIGURE 8:**
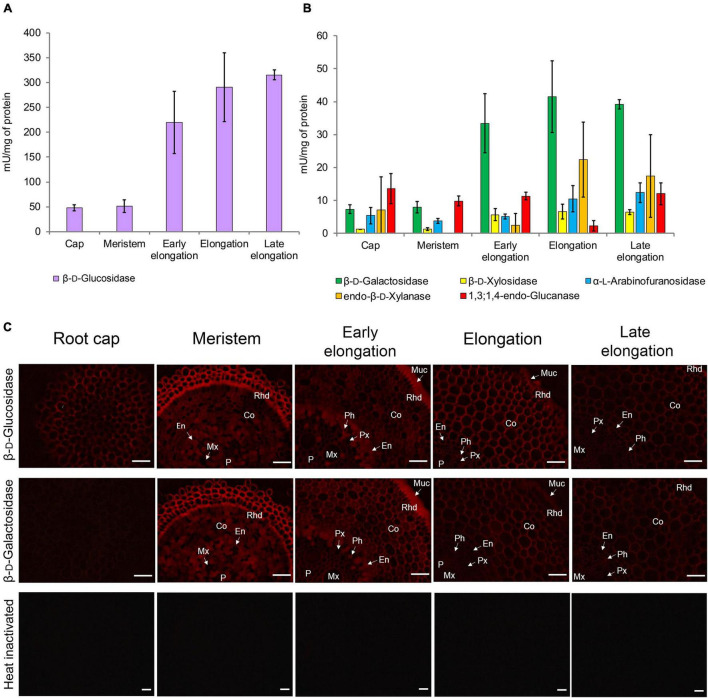
Glycoside hydrolase activities in different zones of maize primary roots assayed *in vitro* and *in situ*. **(A)** The β-D-glucosidase, **(B)** β-D-galactosidase, β-D-xylosidase, α-L-arabinofuranosidase, xylanase, and 1,3;1,4-β-D-glucan endohydrolase activities measured in clarified homogenates. Values are given as mean (*n* = 3) ± SE. **(C)** The β-D-glucosidase and β-D-galactosidase activities of non-fixed maize primary root cross sections. Co, cortex; En, endodermis; Mx, metaxylem; Muc, mucilage; P, pith; Px, protoxylem; Rhd, rhizodermis. Bars are 50 μm.

Glycoside hydrolase (GH) activities in clarified homogenates of maize root segments were assayed by the rate of hydrolysis of corresponding synthetic substrates. The β-glucosidase activity was highest among all the studied enzymatic activities in all root zones (Figure 8A). It was followed by β-D-galactosidase, α-L-arabinofuranosidase (Kozlova et al., 2015), and β-D-xylosidase activities (Figure 8B). The activities of β-glucosidase, β-galactosidase, and β-xylosidase significantly increased from the meristem to the elongation zone and remained at the same level in the late elongation zone (Figures 8A,B). The activity of 1,3;1,4-β-D-glucan endohydrolase (lichenase), in contrast, was constant in all the analyzed root zones except for the active elongation zone where it was significantly reduced (Figure 8B). In clarified homogenates, endo-β-xylanase activity was detected using linear xylopentaose as a substrate being undetectable with xylobiose and xylotriose substrates. Xylanase activity was higher in the active elongation and late elongation zones than in meristem (Figure 8B). We were unable to detect α-L-fucosidase activity in clarified homogenates using *p*-nitrophenyl-α-L-fucopyranoside as a substrate.

β-D-Glucosidase and β-D-galactosidase activities were also analyzed on cross-sections of maize primary root using Res-βDGlc*p* and Res-βDGal*p*, respectively (Figure 8C). In contrast to measurements on clarified homogenates, rates of resorufin release from these two substrates on maize root sections were similar (Figure 8C). For both probes, the highest intensity of fluorescence was observed in meristem and the early elongation zone, and especially in root mucilage. Tissues of the central cylinder displayed lower staining than the cortical part of the maize root (Figure 8C). In contrast to the results of assays with *p*-nitrophenyl glycosides, a visible decrease in staining by resorufin glycosides was observed in the active and late elongation zones. There are two possible explanations for this discrepancy. First, the contribution of cytoplasmic enzymes to total measured activity may grow during the course of elongation growth. Second, the section of the meristem zone included several layers of cells because of their small size, while the elongation and late elongation zone sections contained only one layer where all cells were dissected. Thus, the signal produced by the meristem section was generated by a higher amount of cell walls and cytoplasm than one of elongation and late elongation zones sections. Nevertheless, the high fluorescent signal observed in cell walls after treatment with Res-βDGal*p* and Res-βDGlc*p* indicates that some β-D-galactosidases and β-D-glucosidases are localized in cell walls.

## Discussion

### Metabolic Paths Paved for Different Polysaccharides by GT and GH Expression

#### Xyloglucan

**FIGURE 9:**
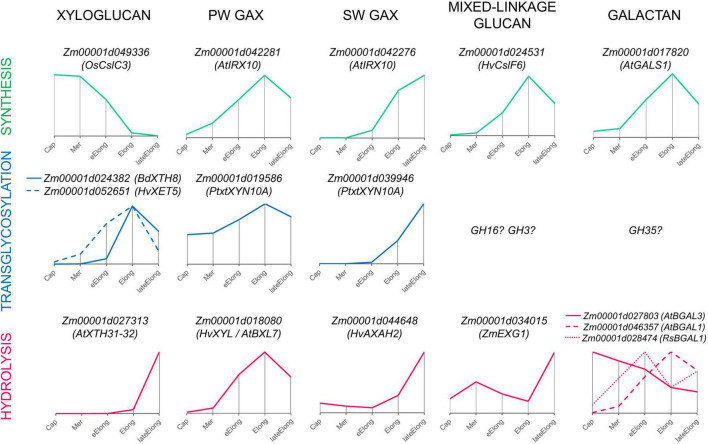
Expression patterns of genes encoding enzymes that can mediate the synthesis, transglycosylation, and hydrolysis of cell wall matrix polysaccharides observed in growing maize roots. Only one gene representing a particular clade or family with the typical dynamics of transcript abundance is shown. The closest characterized homolog for each maize gene is indicated in parenthesis. All profiles are normalized to maximum. Cap, root cap; Mer, meristem; eElong, early elongation zone; Elong, elongation zone; lateElong, late elongation zone; PW, primary cell wall; SW, secondary cell wall.

Enzymes involved in the synthesis of XyGs are actively expressed in the root cap and meristem zone of maize primary root, and the intensity of their transcription is largely reduced in the active and late elongation zones (Figure 9; Kozlova et al., 2020). Transcriptomics and proteomics data on maize primary root suggest that active XyG biosynthesis is followed by intensive rearrangement mediated by XETs in stages of early and active elongation (Figure 9). An exceptionally high level of transcripts in these zones was observed for maize genes homologous to *AtXTH4-5/HvXTH5* and *BdXTH8* (Figure 6). The content of corresponding proteins was also significant. The BdXTH8 protein exhibits predominantly MLG:XyG endotransglucosylase activity and can, thus, produce MLG:XyG covalent bonds (Fan et al., 2018). Moreover, in *Brachypodium*, BdXTH8 accumulates in elongating tissues characterized by high amounts of MLG (Fan et al., 2018). The *Equisetum* homolog of BdXTH8 (EfHTG) is able to use cellulose as the donor substrate and exhibits the dominance of cellulose:XyG endotransglucosylase activity over XyG:XyG homo-transglucosylation activity (Herburger et al., 2021). *HvXTH5* also encodes an XTH capable of using cellulose as the donor substrate although with a rate lower than that for XyG:XyG endotransglycosylation (Hrmova et al., 2007). Homologs of *HvXTH5* in *Arabidopsis* and poplar (*AtXTH4* and *PtxtXTH34*) positively affect the intrusive and expansion growth of fibers and vessels (Nishikubo et al., 2011; Kushwah et al., 2020). The immunolabeling of XyGs was intensive in meristem and the early elongation zone of maize root and gradually decreased in the active and late elongation zones (Kozlova et al., 2020). Because of the high level of gene expression and corresponding protein abundance (Marcon et al., 2015) of putative hetero-transglycosylases in maize primary root, one can suggest that XyG fragments may integrate into the cell wall through covalent linkages with cellulose and MLG during active elongation and, hence, become unrecognizable for antibodies.

Levels of transcripts and proteins for enzymes able to degrade XyGs were much lower than those for XETs (Figures 6, 4). A XEG (GH16), xylan α-L-fucosidase (GH95), and XyG-specific β-D-galactosidase (GH35) were shown to attack XyGs in *Arabidopsis* and *Brachypodium* (Günl et al., 2011; Sampedro et al., 2012; Kaewthai et al., 2013; Rubianes et al., 2019). Maize homologs of these genes had the peak of TGR values in the late elongation zone of maize primary root, and their proteins were accumulated in the late elongation and root hair zones (Figures 4, 6, 9). Thus, we can suggest that after elongation growth is finished XyG molecules can undergo a decrease in molecular weight and backbone decoration.

#### Arabinoxylan

Previously, it has been shown that two sets of GTs putatively involved in GAX synthesis were expressed in maize primary root with different profiles. One of the sets was co-expressed with cellulose-synthases of primary cell walls, while the other was co-expressed with cellulose-synthases for secondary cell walls (Figure 9; Kozlova et al., 2020). Similarly, the two sets of genes encoding AX modifying enzymes can be identified by their expression levels in growing maize roots (Figures 5, 9). One set included genes of GH3 and GH10 enzymes that were co-expressed with genes of primary cell wall biosynthetic machinery (Figure 5A) and similar genes with the highest levels of transcription in early and active elongation zones. Homologs of these genes encode β-D-xylosidases, bifunctional α-L-arabinofuranosidases/β-D-xylosidases, and endo-xylanases in different plants (Figures 3B, 5B). Corresponding activities were detected in clarified homogenates of maize root zones (Figure 8; Kozlova et al., 2015). Thus, it is possible that the action of GHs that remove arabinose as well as xylose substitution is more prominent in the active elongation stage and can be targeted on primary cell wall AXs.

The second set included genes of GH10 and GH51 members that were co-expressed with genes of secondary cell wall biosynthetic machinery (Figures 5A, 9). These genes encode putative xylanases and arabinofuranosidases. Their proteins were found almost exclusively in the stele of the root hair region (Marcon et al., 2015; Figure 5A), suggesting their importance for vascular tissue formation. The maize *WI5* gene, whose significance for this process had already been established in stems (Hu et al., 2020), belonged to the second set. AXs of secondary cell walls may serve as a target for these enzymes.

At least two characterized plant β-xylanases, poplar PtxtXYN10A and papaya CaEXY1, are xylanases-endotransglycosylases (Derba-Maceluch et al., 2015; Johnston et al., 2013) catalyzing both hydrolysis and ligation of xylan chains. It was suggested that the secondary cell wall β-xylanase of poplar acted as transglycosylase and allowed for the release of tensional stress that arises during wall deposition (Derba-Maceluch et al., 2015). Trans-xylosylating activity exceeding the hydrolytic one was reported earlier for *Poacea* seedlings (Johnston et al., 2013) and some other species (Franková and Fry, 2011). The maize roots were characterized by higher endo-trans-xylosylating activity than coleoptiles and leaves (Franková and Fry, 2011). We also found β-xylanase activity in clarified maize homogenates (Figure 8B) but did not test its transglycosylation potential. β-Xylosidases often possess trans-xylosylating activity (Franková and Fry, 2011). Consequently, it is possible that the BXL clade members of GH3 as well as GH10 representatives mediate both degradation and rearrangement of AXs during elongation growth of cells with type II cell walls.

#### Mixed-Linkage Glucan

The dynamics of MLGs during elongation of different cereal organs and features of glucan-degrading enzymes were the most important arguments in favor of the glycoside hydrolase theory of plant cell growth. The β-glucosidase activity of clarified homogenates was at least six times higher than any other one measured within this study (Figures 8A,B). However, most likely, it was provided by cytosolic members of the GH1 family. These enzymes were characterized by an extraordinary transcript and protein abundance (Figure 3). These proteins are accumulated to guarantee the fast release of protective substance DIMBOA from its glucosylated form in case of herbivore attack (Babcock and Esen, 1994). According to the resorufin release from Res-βDGlc by unfixed cross-sections of maize root, the β-glucosidase activity in cell walls was comparable with the β-galactosidase one (Figure 8C). Other members of GH1 with high expression are putative exo-β-mannosidases (Figure 3).

Representatives of the ExoI clade of the GH3 family are more promising candidates for participation in the active turnover of MLGs in cell walls of cereals than GH1 members. J.-B. Kim et al. (2000) purified and characterized ZmEXG1 as the main exo-glucosidase degrading MLGs in maize coleoptiles. We have found a high level of *ZmEXG1* transcripts in maize primary root (Figure 3). Its dynamics were characterized by two peaks, in the meristem and in the late elongation zone, with a significant decrease of transcript abundance in the active elongation zone (Figures 3, 9). Interestingly, the reduction of *ZmEXG1* mRNA abundance in the stage of the most rapid coleoptile growth was also noted by Kim et al. (2000). A similar dynamics of transcript levels were found for two maize homologs of barley ExoI (Figure 3). HvExoI was characterized as the MLG-targeted β-glucosidase in barley seedlings and endosperm (Hrmova et al., 1996, 1998). However, the *Arabidopsis* homolog of *HvExoI* (*AtBGLC1*) was shown to encode the enzyme involved in XyG metabolism. Loss of function of this gene resulted in the accumulation of partially digested XyG fragments in plant tissues (Sampedro et al., 2017). The use of the *Brachypodium* homolog (*BdBGLC1*) of *AtBGLC1* complemented the *Arabidopsis bglc1* mutant (Rubianes et al., 2019). Thus, it remains unclear what polysaccharide is the main target for the GH3 glucosidase activity in maize root cell walls. Moreover, glucosidases of GH3 generally have broad substrate specificity because of their spatial structure (Hrmova et al., 2002). However, it is clear that maize roots have at least three isoforms of exo-β-glucosidases of GH3, which can modify cell wall polysaccharides during the elongation growth of cells. Their specific expression pattern with a decrease of transcript abundance in the zone of the most active elongation requires further studies.

Besides exo-glucosidases, MLGs can be degraded by endo-β-glucanases (Figure 1). We have screened the expression of genes encoding predicted members of the GH9 and GH17 families. In spite of the fact that some known GH9 family members are able to cleave grass hemicelluloses (Yoshida and Komae, 2006), their activity as cellulases or hemicellulases has never been shown *in planta*. Numerous growth defects were described for plants with suppressed gene expression or gene knockout of various GH9 members (Tsabary et al., 2003; Szyjanowicz et al., 2004; Yu et al., 2013); however, no excessive accumulation of any cell wall component in their cell walls were shown. The most extensively studied representative of GH9, KORIGAN, is supposed to serve as part of the cellulose biosynthetic complex (Lampugnani et al., 2019). Genes encoding other proteins of GH9 display a cell-type specific expression (Del Campillo et al., 2004). Their homologs in maize roots were also predominantly expressed in the zones containing these cell types (Supplementary Figure 2). We suggest that in maize, like in many other plant species, GH9 members are rather important for proper assembly of cell walls than associated with the hydrolysis of MLGs.

The other plant protein family of endo-β-glucanases is GH17. It is a huge family that especially develops in grasses (Minic, 2008). Plant proteomes display a wide variety of GH17 members in any physiological state (Nguyen-Kim et al., 2016; Calderan-Rodrigues et al., 2018; Grandis et al., 2019). However, only a few of its representatives were characterized. Some isoforms degrade callose (Benitez-Alfonso et al., 2013), others have antifungal properties and are able to cleave 1,3;1,6-glucans, and some are known to operate with MLGs (Hrmova et al., 2009). However, due to the huge size of the family and the high homology of its members between each other, it is difficult to assume which type of reaction can be catalyzed by a particular isoform. Many representatives of the GH17 family are diversely expressed during the elongation growth of maize roots (Supplementary Figure 3), but their roles in this process require further studies. Nevertheless, the presence of lichenase (endo-β-1,3;1,4-glucanase) activity in clarified homogenates of maize root was established (Figure 8B). Being at the same basic level in all the studied root zones, it was considerably lower in the zone of active elongation. In the same zone, the expression of putative MLG exo-hydrolases also decreased, while the transcript abundance of MLG synthases peaked (Figure 9; Kozlova et al., 2020). MLG degradation accompanies the cessation of elongation growth in maize coleoptiles and internodes (Kim et al., 2000; Zhang et al., 2014) but not in roots (Kozlova et al., 2012, 2020). Proteins for MLG synthases continue to accumulate after the cessation of growth, and their content in the root hair region is higher than that in the elongating part of maize roots (Kozlova et al., 2020). It seems that there are several mechanisms recruited by maize to enhance MLG deposition in cell walls during the stage of the most active elongation.

#### Galactans

Among all tested GH activities, β-galactosidase was the second by its intensity in clarified homogenates after β-glucosidase, though in each zone of maize root, it was at least six times lower than the latter (Figures 8A,B). At the same time, these two activities had comparable intensities while being tested on the maize root cross-sections (Figure 8C). The β-galactosidase activity was found in elongating *Avena* coleoptiles in the 1970s (Johnson et al., 1974). There were also some reports on the reduction of galactan and galactose content in barley and wheat seedlings, oat coleoptiles, and maize roots during elongation growth (Ray, 1963; Dever et al., 1968; Obel et al., 2002; Gibeaut et al., 2005). However, other studies on growing cereal plants reported no significant reduction of 1,4-linked galactose during elongation (Carpita, 1984; Zhang et al., 2014).

Cereal cell walls are thought to have much lower pectin content than cell walls of dicots or non-commelinid monocots (Carpita, 1996). Cell walls in all zones of maize primary roots were labeled by an antibody recognizing β-1,4-galactans (Kozlova et al., 2020), and the maize homolog of *Arabidopsis* galactan-synthase GALS1 was co-expressed with primary cell wall cellulose-synthases (Figure 9; Kozlova et al., 2020). The maize β-galactosidase putatively active toward β-1,4-galactan was also co-expressed with these cellulose-synthases and was characterized by the highest TGR values among all other genes encoding GH35 members (Figure 4). However, several other isoforms of putative pectin-targeted maize BGALs were expressed predominantly in the root cap and early elongation zone (Figure 4). Their homologs in *Arabidopsis* were characterized as galactosidases able to cleave off β-1,4- and β-1,3- but not β-1,6-linked galactose (Gantulga et al., 2008). Interestingly, labeling by the antibody recognizing 1,6-branched 1,4-galactan was much stronger in all zones of maize roots than that of linear galactan (Petrova et al., 2021), and 1,6-branching could serve as the protection for galactan chains against the action of the vast majority of β-galactosidases expressed in maize roots. However, there is another galactosidase of maize whose homolog in radish was shown to degrade 1,6- and 1,3-linked galactosides (Kotake et al., 2005). Its transcript abundance had two peaks: in the early elongation zone and the late elongation zone (Figures 4, 9). In summary, any zone of maize root expresses at least a couple of putative galactosidases able to cleave off any type of terminal galactose residue (Figure 9). The persistence of galactans in maize root cell walls simultaneously with high β-galactosidase activities of homogenates and on sections may be explained by the high rate of galactan turnover during the elongation growth of maize primary roots. Another explanation could be based on the fact that trans-galactosylation reaction often accompanies galactoside hydrolysis (Franková and Fry, 2012). Thus, it is possible that galactans in maize roots are remodeled *in muro* rather than be degraded, assimilated, activated, and returned back to the cell wall.

The importance of galactans for the proper assembly of other polysaccharides has been shown previously for both primary and secondary cell walls on dicots (Moneo-Sánchez et al., 2019, 2020). The mechanisms of this involvement are yet to be studied; however, basic principles can be similar for both type I and type II cell walls.

### Do Glycoside Hydrolases Act as Trans-Glycosylases *in muro*?

Despite a huge pool of information on the existence of various GHs catalyzing the hydrolysis of plant polysaccharides, we suggest that their role is not so straightforward. There are several lines of evidence in support of the activation of transglycosylation reactions catalyzed by GHs *in muro*.

(1) Some GHs, which operate by retaining the substrate anomeric center configuration in the resulting product, can catalyze both hydrolysis and transglycosylation to synthesize oligosaccharides (Davies and Henrissat, 1995). Numerous plant representatives of various GH families able to catalyze the transfer of a glycosyl donor to other than water OH-containing acceptor have been described (GH3: Kim et al., 2000; GH10: Derba-Maceluch et al., 2015; GH35: Franková and Fry, 2012, etc.). The vast amount of plant enzymes, especially cell wall-related, are retaining GHs (Minic, 2008). In this study, the majority of GHs are retaining enzymes; representatives of only three GH families (GH9, GH28, and GH95) operate *via* the inverting mechanism (CAZy database).
(2) In general, the analyzed GHs were characterized by a higher amount of protein than the corresponding GTs. For example, the total protein abundance for two MLG synthases (ZmCslF2 and ZmCslF4) in the elongation zone of maize roots was 498 spectral counts (Kozlova et al., 2020). The abundance of a protein of the known GH3 β-glucosidase (ZmEXG1) active toward MLG (Kim et al., 2000) was 10 times higher and amounted to 4,447 total spectral counts (Figure 3). If we add to this value the protein abundance of numerous endo-β-glucanases expressed in maize roots (Supplementary Figures 2, 3), the lifespan of MLG molecules in the cell wall should be estimated as short. Moreover, many GHs demonstrating higher transcript and protein abundances than their GTs counterparts were found to be co-expressed with them. This can be exemplified by maize homologs of AtGALS1 (Kozlova et al., 2020) and AtBGAL1 (Figures 4, 9), which are supposed to synthesize and degrade, respectively, β-1,4-galactan and belong to one co-expression network. Genes encoding β-xylosidases of GH3, xylanases of GH10, and arabinofuranosidases of GH51 are co-expressed with genes for GTs involved in the synthesis of AXs (Kozlova et al., 2020; Figures 5, 9). Overall, polysaccharide synthesis is a highly demanding process (Barnes and Anderson, 2018). Thus, it seems irrational to break apart (to hydrolyze) the structure immediately after its construction (synthesis).
(3) It is well-documented that GHs catalyze transglycosylation reactions under conditions of high concentration of saccharide substrates (Hrmova and Fincher, 1997; Kim et al., 2000; Franková and Fry, 2012; Derba-Maceluch et al., 2015). In supersaturated solutions of saccharides, transglycosylation reactions catalyzed by glycosidases proceed with higher efficiency because of saccharides competing with water molecules for accommodation in the enzyme active center (MacManus et al., 2001). One can assume that the enzymatic reaction conditions *in muro* and *in vitro* differ in the ratio of sugar and water molecules and, accordingly, in the viscosity of the medium. These are the parameters on which the shift of the thermodynamic equilibrium of hydrolysis:transglycosylation reactions directly depends. Indeed, the water content in plant primary cell walls has been reported to be 60 (Gaff and Carr, 1961) or 80% (Hardegree, 1989). If one assumes that the cell wall contains approximately 30% of saccharides, then, in terms of glucose residues, their content is approximately 1.5 moles, which is obviously higher than the concentration of saccharide substrates in the study on enzymatic catalysis of hydrolysis reaction *in vitro*. For example, all characterized GH3 β-glucosidases catalyzed transglycosylation reactions at substrate concentrations that varied between 2 and 20 mM (Hrmova and Fincher, 1997; Crombie et al., 1998; Kim et al., 2000).
(4) The transglycosylation:hydrolysis ratio with the same substrates varies in different plant organs and species (Franková and Fry, 2011). This indicates that transglycosylation is not just an inevitable side reaction accompanying hydrolysis. The degree of its expression depends on particular enzymes produced by the plant and, hence, can be regulated as being more or less pronounced when it is beneficial for the plant.

The results obtained in this study support the previously suggested physiological importance of GH-mediated transglycosylation for plants (Franková and Fry, 2011). However, the occurrence and details of this process in plants remain to be studied.

## Data Availability Statement

The datasets presented in this study can be found in the BioProject online repository under the accession number PRJNA639682 or using the following link https://www.ncbi.nlm.nih.gov/bioproject/PRJNA639682.

## Author Contributions

LK and TG conceived the study. AN, OG, NP, EE, and LK carried out the experiments and analyzed the results. AN and LK wrote the original draft and prepared all the illustrations, tables, and Supplementary Material. AN, LK, AK, and TG edited the manuscript. All authors reviewed and approved the final version.

## Conflict of Interest

The authors declare that the research was conducted in the absence of any commercial or financial relationships that could be construed as a potential conflict of interest.

## Publisher’s Note

All claims expressed in this article are solely those of the authors and do not necessarily represent those of their affiliated organizations, or those of the publisher, the editors and the reviewers. Any product that may be evaluated in this article, or claim that may be made by its manufacturer, is not guaranteed or endorsed by the publisher.

## Footnotes

1
https://en.novogene.com/

2
http://www.ncbi.nlm.nih.gov/bioproject/639682

3
https://jgi.doe.gov/data-and-tools/bbtools/bb-tools-user-guide/bbduk-guide/

4
https://ensembl.gramene.org/Zea_mays/Info/Index

5
https://www.R-project.org

6
http://www.cytoscape.org

7
http://plants.ensembl.org/index.html

8
http://pfam.xfam.org

9
https://www.ebi.ac.uk/interpro

10
http://www.cbs.dtu.dk/services/SignalP/

11
https://www.ebi.ac.uk/Tools/msa/clustalo/

12
https://phytozome.jgi.doe.gov/pz/portal.html

13
https://www.uniprot.org/

14
https://www.ncbi.nlm.nih.gov/protein/

15
http://www.cazy.org
