# Supplementary material for: Forgotten Actors: Glycoside Hydrolases During Elongation Growth of Maize Primary Root
Source: Front Plant Sci. 2022 Feb 10;12:802424. doi: 10.3389/fpls.2021.802424 (PMC8866823; doi:10.3389/fpls.2021.802424)
Supplement: Supplementary file 1 [file Data_Sheet_1.docx]

**Supplementary File 1.** The phylogenetic analysis and gene expression of GH1, GH9, GH17, GH28 families’ members in maize.


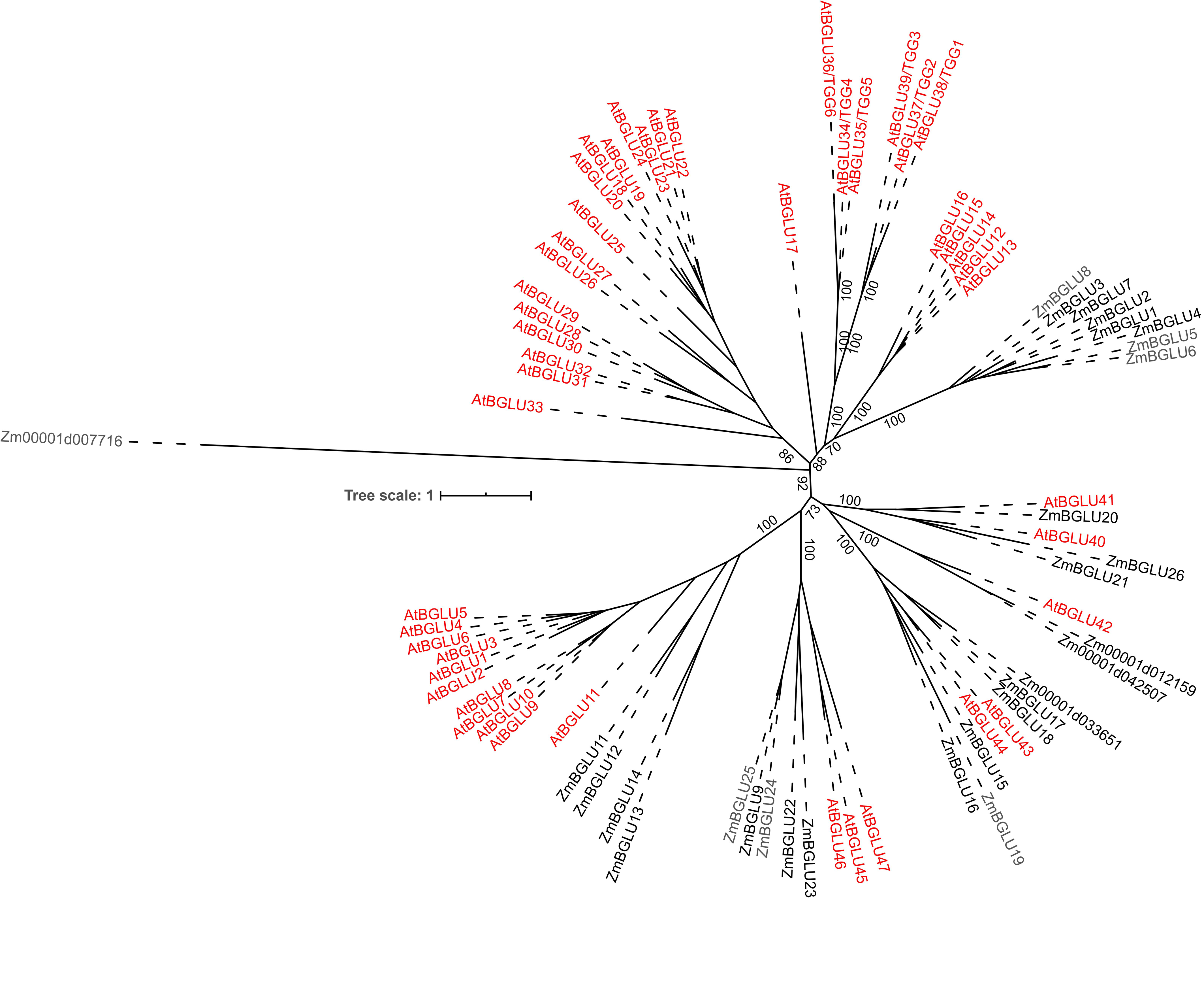


**Supplementary Figure 1.** The unrooted maximum likelihood phylogenetic tree of plant GH1 protein family members. The maize expressed genes are given in black, and non-expressed (TGR values lower than 16 in all analyzed root samples) in grey, *Arabidopsis thaliana* genes in red. *A. thaliana* gene names follow Xu et al. (2004), *AtSFR2* gene follows Thorlby et al. (2004), maize gene names follow Gómez-Anduro et al. (2011). Numbers indicate the ultrafast bootstrap support values for some branches.


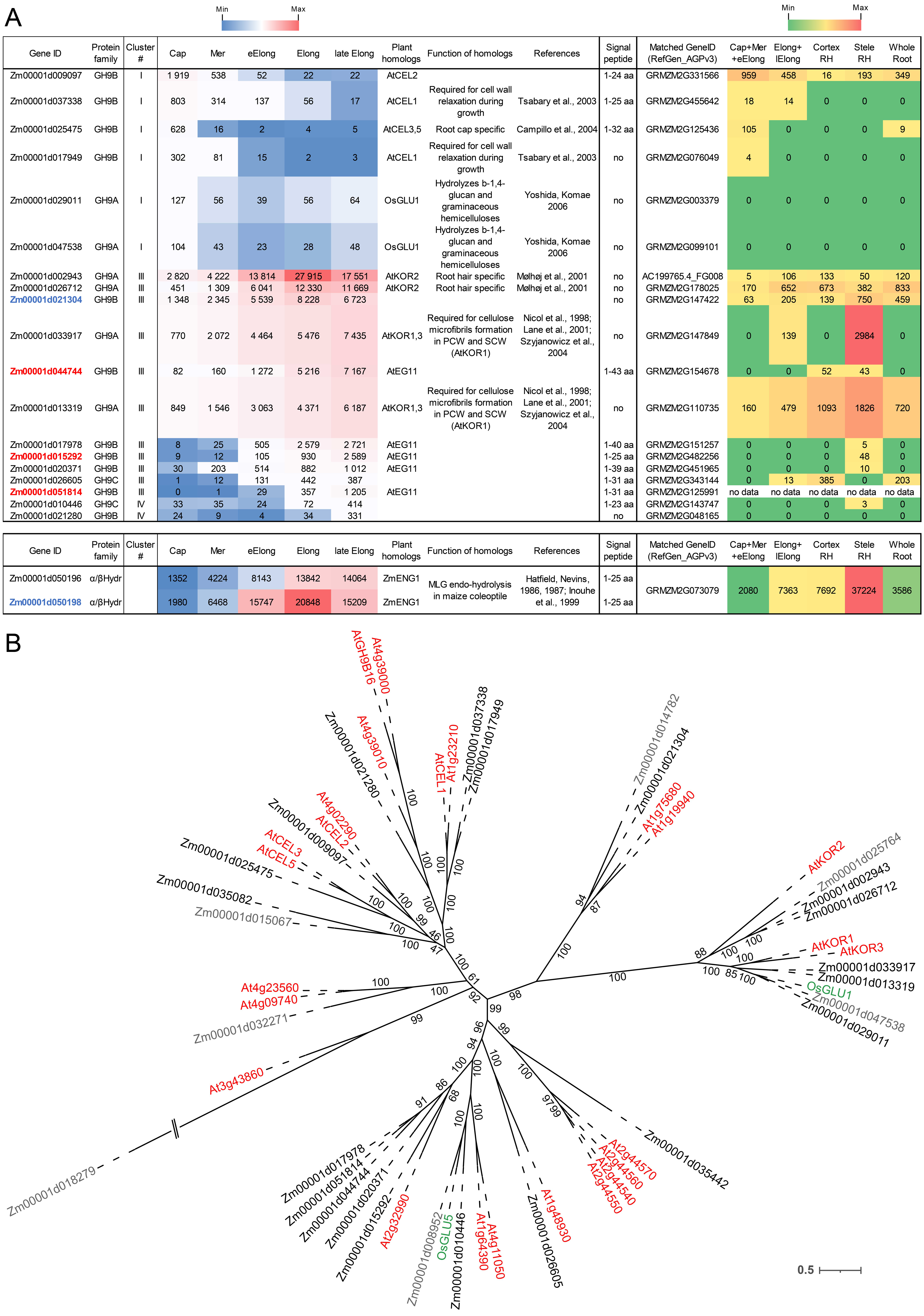


**Supplementary Figure 2.** The expression of maize genes encoding putative GH9 β-d-glucanases in maize root and phylogenetic analysis of plant GH9 family. (**A**) The level of transcripts (TGR, red-blue heat map) and abundance of corresponding protein (averaged and normalized total spectral counts (Marcon et al., 2015), red-green heat map) of genes encoding putative β-d-glucanases of the GH9 protein family in the analyzed maize root zones. The genes with expression values below 100 in all studied samples are not shown. TGR values are sorted from maximum to minimum within each cluster. Maize genes that co-expressed with the primary and secondary cell wall cellulose-synthases are given in blue and red, respectively. The GH9A – the protein family members that possess both cytosolic and transmembrane domain, GH9B – members that have signal peptide in protein sequence, and GH9C – members that have both signal peptide and CBM49 (PF09478) domain. Cap – root cap, Mer – meristem, eElong – early elongation zone, Elong – zone of active elongation, lateElong – zone of late elongation before root hair initiation, RH – root hair zone, no data – no corresponding peptides were found by Marcon et al. (2015) proteomic analysis. PCW – primary cell wall, SCW – secondary cell wall, MLG – mixed-linkage glucan. **(B)** Unrooted maximum likelihood phylogenetic tree of the plant GH9 protein family members. The maize expressed genes are given in black, and non-expressed (TGR values lower than 16 in all analyzed root samples) in grey, *Arabidopsis thaliana* genes in red, and rice in green (only genes encoding enzymes with shown enzymatic activity are shown (Yoshida, Komae, 2006). *A. thaliana* gene names follow the references in **(A)**. Numbers indicate the ultrafast bootstrap support values for some branches.


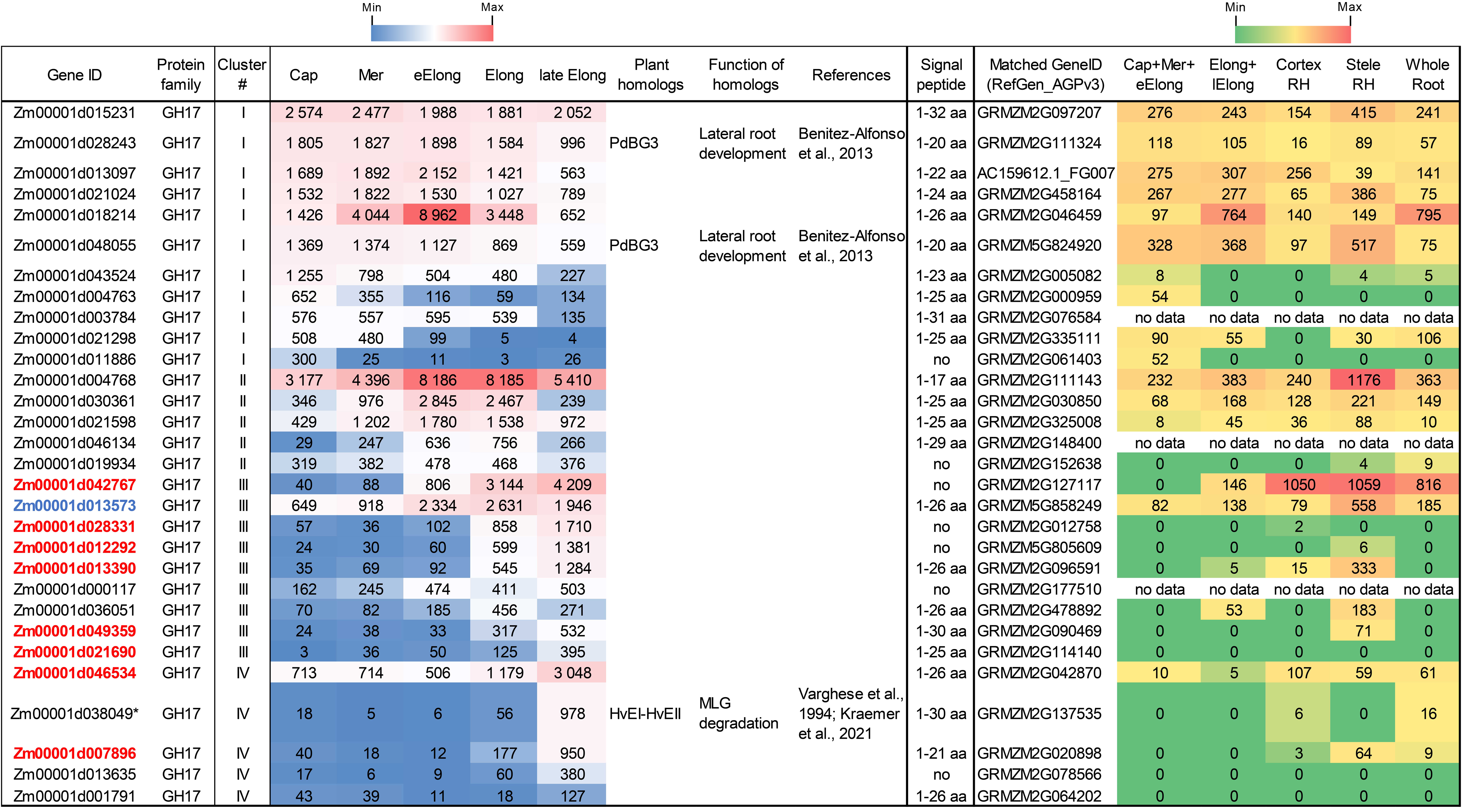


**Supplementary Figure 3.** The expression of maize genes encoding the putative GH17 β-d-glucanases in maize root. The level of transcripts (TGR, red-blue heat map) and abundance of the corresponding protein (averaged and normalized total spectral counts (Marcon et al., 2015), red-green heat map) of genes encoding putative β-d-glucanases of the GH17 protein family in various zones of maize root. The genes with expression values below 100 in all studied samples are not shown. TGR values are sorted from maximum to minimum within each cluster. Maize genes co-expressed with the primary and secondary cell wall cellulose-synthases are given in blue, and red, respectively. Cap – root cap, Mer – meristem, eElong – early elongation zone, Elong – zone of active elongation, lateElong – zone of late elongation before root hair initiation, RH – root hair zone, MLG – mixed-linkage glucan, no data – no corresponding peptides were found by Marcon et al. (2015) proteomic analysis.


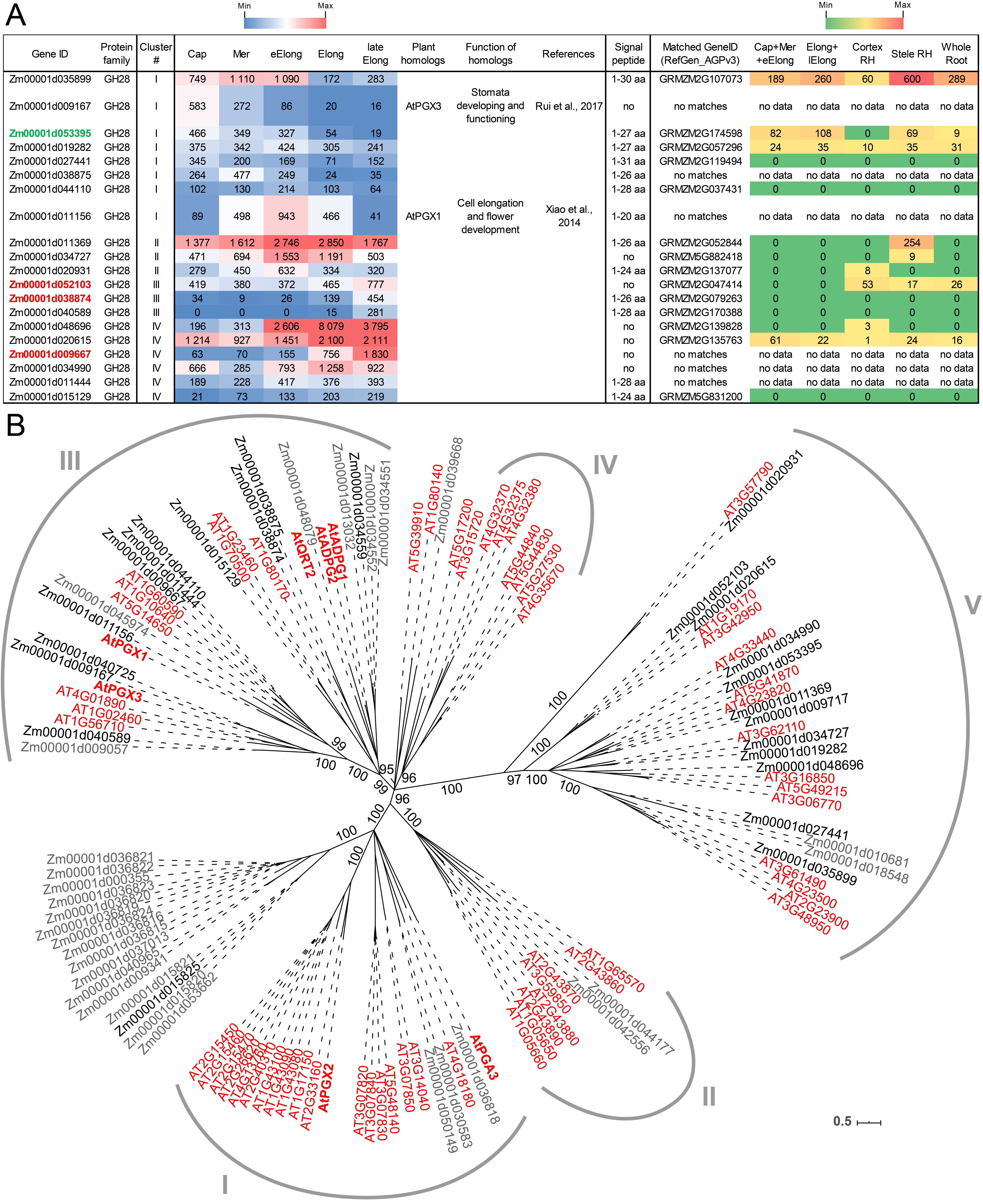


**Supplementary Figure 4.** The expression of maize genes encoding putative GH28 polygalacturonases in the maize root and phylogenetic analysis of plant GH28 family. (**A**) The level of transcripts (TGR, red-blue heat map) and abundance of corresponding protein (averaged and normalized total spectral counts (Marcon et al., 2015), red-green heat map) of the genes encoding putative polygalacturonases of GH28 protein family in the analyzed maize root zones. The genes with expression values below 100 in all studied samples are not shown. TGR values are sorted from maximum to minimum within each cluster. Maize genes co-expressed with the primary and secondary cell wall cellulose-synthases are given in blue and red, respectively. Cap – root cap, Mer – meristem, eElong – early elongation zone, Elong – zone of active elongation, lateElong – zone of late elongation before root hair initiation, RH – root hair zone, no data – no corresponding peptides were found by Marcon et al. (2015) proteomic analysis, no matches – no gene models matching to the AGPv4 maize genome assembly. PCW – primary cell wall, SCW – secondary cell wall, MLG – mixed-linkage glucan. **(B)** The unrooted maximum likelihood phylogenetic tree of plant GH28 protein family members. The maize expressed genes are given in black, and non-expressed (TGR values lower than 16 in all analyzed root samples) in grey, the *Arabidopsis thaliana* genes in red. The clade numbering follows Cao (2012). Numbers indicate the ultrafast bootstrap support values for some branches.

**References**

Cao, J. (2012). The pectin lyases in *Arabidopsis thaliana*: evolution, selection and expression profiles. *PLoS ONE 7*(10): e46944. https://doi.org/10.1371/journal.pone.0046944

Gómez-Anduro, G., Ceniceros-Ojeda, E. A., Casados-Vázquez, L. E., Bencivenni, C., Sierra-Beltrán, A., Murillo-Amador, B., et al. (2011). Genome-wide analysis of the beta-glucosidase gene family in maize (*Zea mays* L. var B73). *Plant Mol. Biol*. 77, 159–183.

Marcon, C., Malik, W. A., Walley, J. W., Shen, Z. X., Paschold, A., Smith, L. G., et al. (2015). A High-Resolution Tissue-Specific Proteome and Phosphoproteome Atlas of Maize Primary Roots Reveals Functional Gradients along the Root Axes. *Plant Physiol*. 168, 233-+. doi:10.1104/pp.15.00138.

Thorlby, G., Fourrier, N., and Warren, G. (2004). The SENSITIVE TO FREEZING2 gene, required for freezing tolerance in *Arabidopsis thaliana*, encodes a β-glucosidase. *Plant Cell* 16, 2192–2203.

Xu, Z., Escamilla-Treviño, L., Zeng, L., Lalgondar, M., Bevan, D., Winkel, B., et al. (2004). Functional genomic analysis of *Arabidopsis thaliana* glycoside hydrolase family 1. *Plant Mol. Biol*. 55, 343–367.

Yoshida, K., and Komae, K. (2006). A rice family 9 glycoside hydrolase isozyme with broad substrate specificity for hemicelluloses in type II cell walls. *Plant cell Physiol*. 47, 1541–1554.
